# Accuracy of Human Papillomavirus (HPV) Testing on Urine and Vaginal Self-Samples Compared to Clinician-Collected Cervical Sample in Women Referred to Colposcopy

**DOI:** 10.3390/v15091889

**Published:** 2023-09-07

**Authors:** Marianna Martinelli, Chiara Giubbi, Maria Letizia Di Meo, Federica Perdoni, Rosario Musumeci, Biagio Eugenio Leone, Robert Fruscio, Fabio Landoni, Clementina Elvezia Cocuzza

**Affiliations:** 1 Department of Medicine and Surgery, University of Milano-Bicocca, 20900 Monza, Italy; chiara.giubbi@unimib.it (C.G.); federica.perdoni@unimib.it (F.P.); rosario.musumeci@unimib.it (R.M.); biagioeugenio.leone@unimib.it (B.E.L.); robert.fruscio@unimib.it (R.F.); fabio.landoni@unimib.it (F.L.); clementina.cocuzza@unimib.it (C.E.C.); 2 IRCCS San Gerardo dei Tintori, 20900 Monza, Italy; marialetizia.dimeo@gmail.com

**Keywords:** HPV infection, self-sampling, HPV testing, cervical cancer, low-risk HPV

## Abstract

In the context of cervical cancer prevention, where human papillomavirus (HPV) infection is pivotal, HPV testing is replacing Pap Smear in primary screening. This transition offers an opportunity for integrating self-sampling to enhance coverage. We evaluated the accuracy of HPV testing using self-collected urine and vaginal samples, comparing them to physician-collected cervical swabs. From a cohort of 245 women with abnormal cytology, we collected self-sampled vaginal, urine, and clinician-administered cervical specimens. Employing Anyplex™II HPV28 assay, outcomes revealed HPV positivity rates of 75.1% (cervical), 78.4% (vaginal), and 77.1% (urine). Significant, hr-HPV detection concordance was observed between self-taken cervical samples and clinical counterparts (k = 0.898 for vaginal; k = 0.715 for urine). This study extends beyond accuracy, highlighting self-collected sample efficacy in detecting high-grade cervical lesions. The insight underscores self-sampling’s role in bolstering participation and aligns with WHO’s goal to eliminate cervical cancer by 2030.

## 1. Introduction

Cervical cancer (CC) is one of the most common cancers in women and ranks fourth among female malignancies (global incidence rate standardized by age equal to 13.3 out of 100,000 women) according to GLOBOCAN 2020 data [1]. Less developed countries represent the areas with the highest prevalence and mortality rates, particularly in Central and Eastern Africa. In Italy, the age-standardized incidence rate of CC is 6.9 per 100,000 women [2], with 2400 new cases in 2020 [3].

Analogous to its relevance in other neoplastic conditions, screening has emerged as a pre-eminent strategy for mitigating the impact of cervical carcinoma on the populace. Since the inception of the Pap test in the 1950s, the conspicuous effectiveness of preventive screening has rapidly manifested in the confrontation against cervical cancer. However, the outcomes of cytological examination are contingent upon the expertise of medical pathologists for interpretation.

Human papillomavirus (HPV), an established DNA virus, assumes a pivotal role as a requisite factor in the genesis of cervical cancer. Consequently, the emergence of methodologies proficient in detecting the presence of viral DNA specific to high-risk HPV (hr-HPV) variants has furnished supplementary tools to amplify the efficiency of screening initiatives, engendering heightened clinical sensitivity compared to the Pap test [4,5]. The Italian 2014–2018 National Prevention Plan radically endorsed the integration of the HPV test within cervical screening protocols as a primary modality, progressively superseding the Pap test or serving as a discernment criterion during the transitional phase for instances of marginal cytological anomalies. The 2020–2025 National Prevention Plan further focalizes on culminating the transition to primary screening grounded upon the HPV-DNA test. Additionally, it endeavors to delineate cervical cancer screening algorithms meticulously tailored for women who have undergone HPV vaccination.

There are several different commercially available HPV tests already validated following the Meijer or VALGENT protocols [6,7,8]. All validated tests should demonstrate non-inferior sensitivity and specificity to detect Cervical Intraepithelial Neoplasia grade 2 (CIN2+) or more compared with a defined comparator test [8]. All these validations are important to standardize the results to obtain comparable diagnostic outcomes and to collect equivalent data to monitor HPV infection spread and related diseases. Optimal pre-analytic and analytic approaches are essential in order to detect the presence of different HPV genotypes, to have an accurate quantification of viral genomes, and to detect the presence of mRNA, which may useful biomarkers for a reliable evaluation of cancer risk [9,10].

One of the major obstacles in implementing efficient prevention programs is to ensure the regular participation of women at the highest risk for cervical cancer development (age range of 25–65 years). In the context of cervical cancer screening in Italy, the data derived from surveillance efforts reveals that several emerging factors contribute to non-adherence to screening protocols. These factors encompass the lack of comprehensive information, a disposition towards indolence, and constraints imposed by limited time availability. Moreover, women who abstain from screening have communicated additional concerns, including feelings of embarrassment and apprehension linked to the prospect of physician examination [11]. The prospective implementation of self-sampling methodologies presents an auspicious avenue that can be used to address the challenge of engaging women who remain underserved by screening initiatives or have never undergone such procedures [12,13]. Notably, the application of HPV testing on self-collected samples has garnered attention due to its demonstrated parity in accuracy when compared to cervical samples procured by clinicians. This method exhibits commendable sensitivity in detecting early cervical pre-cancer lesions. Beyond its diagnostic efficacy, this approach holds the promise of mitigating socio-cultural barriers intrinsic to gynecological examinations, thereby potentially enhancing screening participation rates among previously underserved cohorts [14,15].

The collection of vaginal specimens allows HPV detection because most of detached cervical cells are released with mucous in the vagina. Vaginal self-collected sampling using a vaginal swab is easy to execute and could be performed at home. Another sample that could be used for self-sampling is first-void urine (FVU), the collection of which is non-invasive and has showed similar results in HPV testing compared to cervical specimens [16,17]. During urination, the mucous and debris of cells from the cervix and uterus are captured by urine flow, explaining the presence of HPV DNA in urine samples [18]. Several devices for vaginal and urine self-collection are commercially available and well accepted by women [19,20,21].

Therefore, the aim of this study is to evaluate the accuracy of HPV DNA testing conducted on urine and vaginal self-samples compared to clinician-collected cervical samples and to investigate the distribution of HPV genotypes (both high- and low-risk) in different sample types.

## 2. Materials and Methods

### 2.1. Ethics Statement

The study protocol was approved by the Ethics Committee of University of Milano-Bicocca (protocol numbers 0037320/2017 and 0086409/2018). All subjects provided written informed consent to participate in the study.

### 2.2. Study Design and Sample Collection

A cohort comprising 245 women with a recent diagnosis of cervical dysplasia were enrolled at the Colposcopy Clinic of IRCSS San Gerardo dei Tintori, Monza, Italy, during the period spanning from May 2017 to January 2021. All women were adequately informed about the study by Colposcopy Clinic staff and were asked to autonomously collect a urine specimen (20 mL) (Colli-pee^®^, Novosanis, Wijnegem, Belgium) and a vaginal self-sample (FLOQSwab^®^, Copan Italia Spa, Brescia, Italy) before colposcopy examination. An information brochure of the study and instructions on how to use the devices were given to all participants.

Immunocompromised patients, women with autoimmune diseases or any diseases involving the immune system, patients with HIV infection, patients with a presumed or confirmed pregnancy, patients with a diagnosis of any malignancies, and patients who are undergoing or have finished a course of chemotherapy in the six months preceding the enrollment were excluded from the study.

During the colposcopy examination, the gynecologist collected a cervical sample using an L-shaped Endocervical/Esocervical FLOQSwab^®^ (Copan Italia Spa, Brescia, Italy) and the Pap smear test was repeated if it had been performed over 6 months before the date of the colposcopy. A cytological diagnosis of cervical lesions was performed at the hospital, and findings were assessed according to the 2001 Bethesda System for cervical cytological reporting. Cytological findings were classified as follow: HSIL (high-grade squamous intraepithelial lesion); ASC-H (atypical squamous cells, cannot exclude HSIL); LSIL (low-grade squamous intraepithelial lesion); ASC-US (atypical squamous cells of undetermined significance); AGC (atypical glandular cells); and NILM (negative for intraepithelial lesion or malignancy). Women underwent biopsy examination or conization treatment depending on the colposcopy results. Histological findings were classified as cervical intraepithelial neoplasia grade 1, 2, or 3 (CIN 1, 2, or 3); squamous cell carcinoma; adenocarcinoma; or adenosquamous carcinoma. The classification of histological findings was conducted according to the WHO histological classification of tumors [22]. 

All physician-collected cervical, vaginal, and urine samples were tested at the Clinical Microbiology Laboratory of the Department of Medicine and Surgery, University of Milano-Bicocca, Italy.

### 2.3. Pre-Analytical Sample Processing

Cervical samples were collected using the L-shaped Endocervical/Esocervical FLOQSwab^®^ and transported in a tube with 20 mL of ThinPrep^®^ PreservCyt^®^ Solution (HOLOGIC^®^, Marlborough, MA, USA). All samples were well shaken using vortex for 30 s and 1.5 mL aliquots were made. One aliquot was used for DNA extraction purposes.

Vaginal self-samples were transported dry to the laboratory and immediately suspended in 5mL of ThinPrep^®^ PreservCyt^®^ Solution. Five aliquots were made (one was used for DNA extraction and the others were stored at −20 °C).

After arriving to the laboratory, urine specimens were shaken for 30 s using the vortex and 1.5 mL aliquots were made. One aliquot was used for nucleic acid extraction, while the others were stored at −20 °C.

### 2.4. Nucleic Acid Extraction

For 180 patients, DNA isolation was performed starting from 1 mL of each specimen using the NucliSENS^®^ easyMag^®^ (bioMérieux, Marcy-l’Étoile, France), an automated system used for isolating nucleic acid from clinical samples. The protocol chosen was “specific B”, characterized by a higher final elution temperature (about 70 °C), and silica beads (diluted in a ratio of 1:2) were used, in accordance with the manufacturer’s instructions. The nucleic acids extracted from cervical and vaginal samples were eluted in 100 μL of NucliSENS^®^ easyMag^®^ elution buffer (bioMérieux, Marcy-l’Étoile, France), and the ones from urine were eluted in 40 μL.

Samples from the remaining 65 women were extracted using the STARMag 96 × 4 Universal Cartridge Kit (Seegene, Seoul, Republic of Korea) on Microlab Nimbus, a totally automated platform for nucleic acid extraction and PCR plate preparation. The starting volume used for the extraction was 200 µL, and the elution volume was 100 µL for all the three different sample’s types.

Cervical, vaginal, and urine samples obtained from 50 women were analyzed using both nucleic acid extraction methods to evaluate the concordance of the results obtained following different protocols.

### 2.5. HPV DNA Detection

Cervical, vaginal, and urine specimens underwent HPV typing using a commercial kit known as Anyplex™II HPV28 (Seegene, Seoul, Republic of Korea). This kit is proficient in identifying 19 high-risk HPV types (16, 18, 26, 31, 33, 35, 39, 45, 51, 52, 53, 56, 58, 59, 66, 68, 69, 73, and 82) and 9 low-risk HPV types (6, 11, 40, 42, 43, 44, 54, 61, and 70) with two different real-time PCR reactions performed on CFX96 (Bio-Rad, Hercules, CA, USA) as reported by the manufacturer’s instructions. DNA detection was performed with 5 μL of DNA in each of the two 20 μL reaction mixtures with two different primer sets. Data recording and interpretation were performed with Seegene Viewer software, in accordance with the manufacturer’s instructions.

### 2.6. Statistical Analysis

Qualitative and quantitative variables were summarized with absolute (relative) frequencies and medians (interquartile ranges, IQRs), respectively.

The HPV typing analysis was performed considering as high-risk types: HPV 16, 18, 31, 33, 35, 39, 45, 51, 52, 56, 58, 59, 66, and 68 and low-risk types: HPV 6, 11, 26, 40, 42, 43, 44, 53, 54, 61, 69, 70, 73, and 82.

The agreement of HPV typing results between paired cases was evaluated with Cohen’s kappa (κ) statistics and their uneven distribution was evaluated with McNemar’s test using GraphPad QuickCalcs software (updated in 2014, available at http://graphpad.com/quickcalcs). Agreement was interpreted as slight (k < 0.200), fair (0.200 < k < 0.401), moderate (0.400 < k < 0.601), good (0.600 < k < 0.801), very good (0.800 < k < 1.000), and perfect (k = 1.000) [23].

## 3. Results

### 3.1. Study Participants

Cervical, vaginal, and urine samples collected from 245 women were analyzed. The average age of the patients at the time of enrollment in the study was 38.6 years (range: 17–67 years), while the median was 38 years (IQR: 31–46 years). Abnormal Pap smears at the enrolment were detected in 92.6% (227/245) of the study participants with low-grade lesions, with ASC-US (23.3%; 57/245) and LSIL (43.7%; 107/245) being the most frequently reported. Data regarding cytological lesions are reported in Table 1. According to colposcopy results, 60 (24.5%; 60/245) women underwent biopsy and, among these, 47 (78.3%; 47/60) were treated by conization.

### 3.2. Comparison of the Two Different Nucleic Acid Extraction Methods

**Figure 1:**
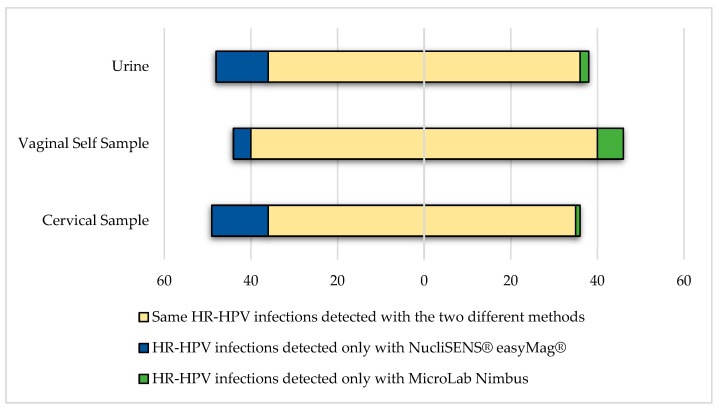
hr-HPV DNA detection using the two different nucleic acid extraction protocols.

Samples collected from 50 women were randomly chosen and analyzed to compare results obtained using the two different nucleic acid extraction protocols. The concordance was evaluated considering just the positivity for hr-HPV genotypes. A very high percentage of hr-HPV positivity was obtained among all types of samples analyzed and using both extraction methods. In particular, using the NucliSENS^®^ easyMag^®^ extraction protocol, we observed hr-HPV positivity rates of 80% (40/50), 78% (39/50), and 76% (38/50) in cervical, vaginal, and urine samples, respectively. We obtained the following results using the platform MicroLab Nimbus: 72% (36/50), 80% (40/50), and 70% (35/50) in cervical, vaginal, and urine samples, respectively. A good concordance in hr-HPV detection was observed using the two different extraction protocols by comparing the results obtained from all three types of samples analyzed, as reported in Figure 1 (k = 0.747, k = 0.940, and 0.783 for urine, vaginal, and cervical samples, respectively)

### 3.3. HPV DNA Detection 

**Figure 2:**
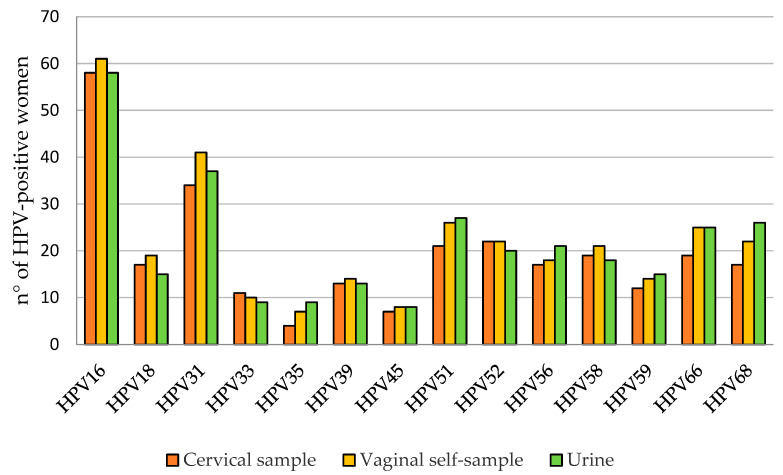
Prevalence of different high-risk HPV genotypes.

**Figure 3:**
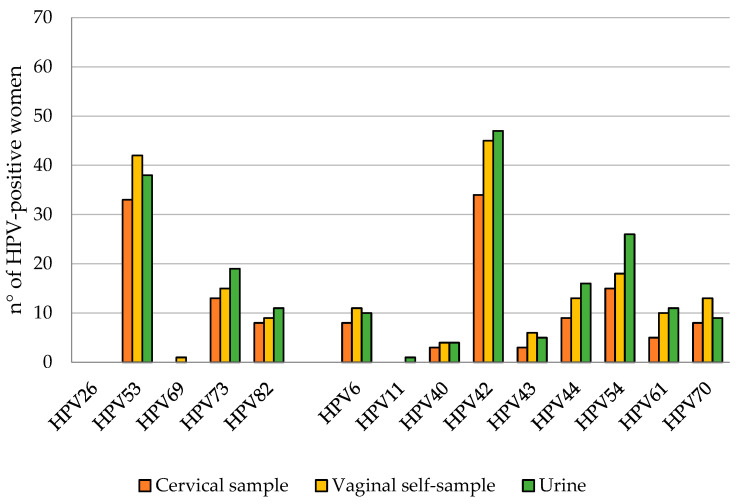
Prevalence of different low-risk HPV genotypes.

Overall, 75.1% (184/245), 78.4% (192/245), and 77.1% (189/245) of cervical, vaginal, and urine samples, respectively, were positive for at least 1 of the 28 HPV genotypes detected using the Anyplex™ II HPV28 Detection Kit. In particular, 161 women (65.7%) were positive for at least one hr-HPV both in cervical and urine samples, while 170 women (69.4%) were positive in vaginal self-samples. lr-HPV infection was detected in 40.0% (98/245) of the cervical samples, 48.6% (119/245) of the vaginal self-samples, and 50.2% (123/245) of the urine samples. HPV16 and HPV31 were the most frequently reported hr-HPV types (Figure 2), while HPV53 and HPV42 were the most reported among lr-HPV (Figure 3). The adenosquamous carcinoma and the adenocarcinoma detected in two of the study participants were HPV16- and HPV18-positive, respectively.

As shown in Table 2, the percentage of women with hr-HPV infection in the three different specimens was higher in the presence of more severe cytological lesions (77.8%, 81.5%, and 77.8% in cervical, vaginal, and urine samples, respectively). In women with low-grade cervical dysplasia, the percentages were 65.2%, 68.3%, and 65.9% and in those without lesions, the percentages were 27.8%, 27.8%, and 22.2%.

Table 3 reports the HPV positivity rates among the subgroup of 60 women who underwent biopsy and/or conization based on colposcopy findings. Forty-four women were diagnosed as CIN2+ and 42 (95.5%), 42 (95.5%), and 40 (90.9%) were hr-HPV-positive in cervical, vaginal, and urine specimens, respectively. Among the women diagnosed as CIN2-, the hr-HPV positivity rate dropped to 68.8% in all sample types.

**Figure 4:**
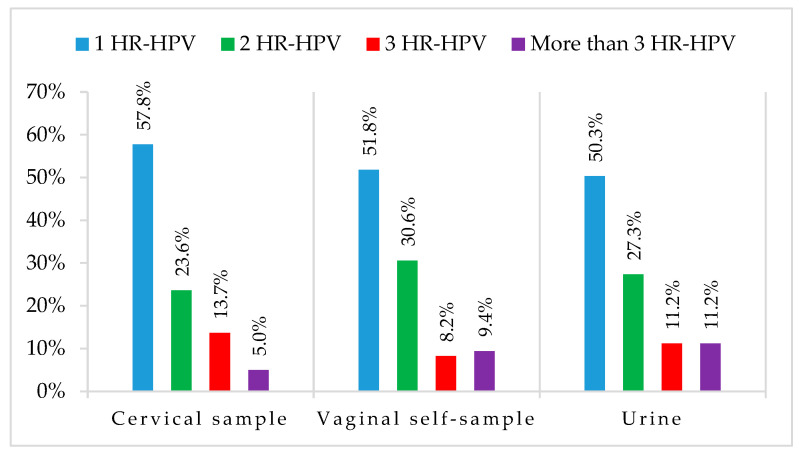
Multiple hr-HPV infections in cervical, vaginal, and urine samples.

Almost half of hr-HPV-positive samples were coinfected by more than one hr-HPV genotype, with urine and vaginal self-samples showing more multiple cervical sample infections: 49.7% (80/161), 48.2% (82/170), and 42.2% (68/161), respectively (Figure 4).

### 3.4. Agreement between Cervical Samples and Vaginal Self-Samples

**Figure 5:**
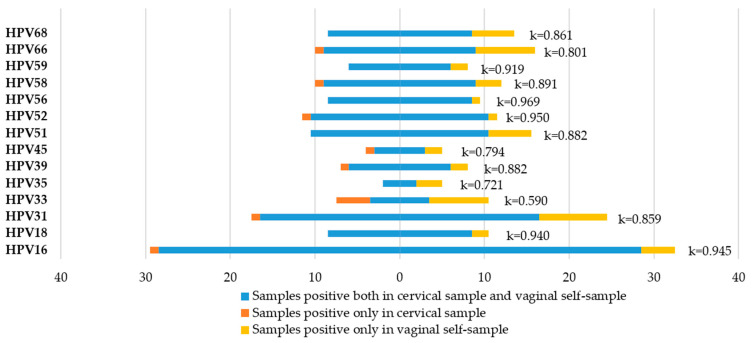
hr-HPV-type-specific agreement between cervical and vaginal self-samples.

Considering the positivity for at least one of hr-HPV, the observed agreement between cervical and vaginal samples was 95.5% with k = 0.898. HPV16 and HPV18, which are the main responsible for cervical cancer, showed a very good agreement with k = 0.945 and k = 0.940, respectively. The concordance in lr-HPV detection was 88.2% with k = 0.762. Concordance in the hr-HPV test results between cervical and vaginal specimens is shown in Figure 5. Data on the accuracy of vaginal self-specimens for HPV detection are reported in Table 4.

### 3.5. Agreement between Cervical Samples and Urine Samples

**Figure 6:**
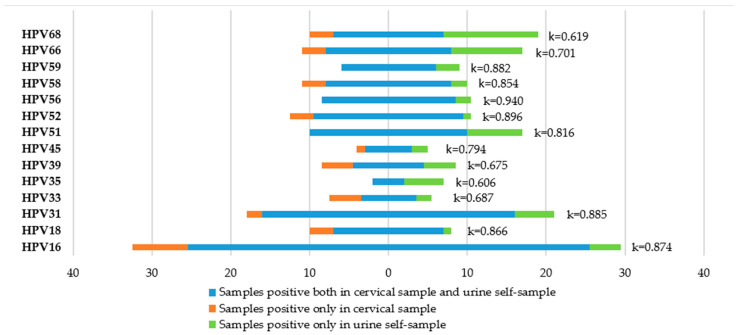
hr-HPV-type-specific agreement between cervical and urine samples.

The concordance rates between cervical and urine specimens were 90.6% (with k = 0.792) for hr-HPV and 85.7% (with k = 0.715) for lr-HPV. In particular, a very good concordance rate in HPV16 (k= 0.874) and HPV18 (k = 0.866) results was found. Genotype-specific HPV agreement rates are reported in Figure 6. Data on the accuracy of urine samples for HPV detection are reported in Table 5.

### 3.6. Accuracy of CIN2+ Detection

Table 6 and Table 7 show data about specificity and sensitivity rates for the hr-HPV detection of self-collected samples respect to cervical specimens for the detection of CIN2+. Women with <CIN2 histological results were considered healthy cases. Notably, sensitivity rates for CIN2+ detection were remarkably high. Cervical and vaginal specimens were equally sensitive for CIN2+ detection (95.5%), while the sensitivity of urine was slightly inferior (90.9%). On the other hand, specificity was demonstrated to be the same with the three different specimens when only women with histological diagnosis were analyzed, while it was inferior in vaginal specimens when all samples were studied. When restricting the analysis to a subset of seven of the hr-HPV samples most related to cervical cancer (HPV16, 18, 31, 33, 45, 52, and 58), 122, 134, and 119 women were hr-HPV-positive in cervical, vaginal, and urine specimens, respectively. When considering only these seven hr-HPV genotypes, lower clinical sensitivity rates for the detection of CIN2+ lesions were observed, corresponding to 90.9%, 93.2%, and 84.1% rates in the three sample types, respectively.

## 4. Discussion

According to the World Health Organization, without any additional actions, the annual number of new cases of cervical cancer will rise from 570,000 to 700,000 between 2018 and 2030. In order to eliminate cervical cancer as a public health problem, one of the targets to reach by 2030 is to screen 70% of women with a high-performance test by 35 years of age and again by 45 years of age [24]. Over the last few years, different countries are switching to hr-HPV testing for cervical cancer screening, at least in women above the age of 30, because HPV-based screening has a 60–70% better protection ability against invasive cervical cancer when compared to cytology [5,25,26]. The use of molecular methods based on nucleic acid amplification to detect a specific pathogens in biological samples is commonly used in diagnostic laboratories. To perform an accurate analysis, it is important to define an adequate sample volume to obtain a high enough amount of DNA or RNA and to remove components of microorganisms or cellular materials which may inhibit amplification reactions. Different studies have already shown that various tests could lead to different results or dissimilar genotyping identification methods due to different primer pairs used, HPV genes chosen as amplification targets, or reaction parameters set up for testing [6,27,28]. On the contrary, limited studies have focused their attention to the influence of pre-analytic procedures on HPV testing results and genotyping [29]. For example, Donà and colleagues have compared three different extraction procedures using two diverse PCR-based HPV genotyping tests and they found different percentages of concordant results considering manual vs. automated procedures which were also influenced by the sample starting volume used to obtain the DNA extracted. In fact, different starting volumes in association with different extraction methods could lead to a different amount of nucleic acid useful as a template for the amplification reaction [29]. In this study, we compared the impact of two different pre-analytic processes to be used for HPV detection in cervical, vaginal, and urine samples. In particular, samples were extracted using two extraction protocols using two instruments, NucliSENS^®^ easyMag^®^ and Seegene Microlab Nimbus platforms, implemented with the STARMag 96X4 Universal Cartridge Kit. A good analytical concordance was observed (k = 0.747, k = 0.940, and 0.783 for urine, vaginal, and cervical samples, respectively); however, slightly higher hr-HPV positivity was observed among aliquots extracted using NucliSENS^®^ easyMag^®^, probably in part resulting from the different starting volumes: 1 mL for NucliSENS^®^ easyMag^®^ compared to 200 µL for the STARMag 96X4 Universal Cartridge Kit protocol on Microlab Nimbus. The clinical relevance of these differences in HPV detection is still not fully understood due to the low number of samples analyzed.

In this study, we also compared the accuracy of the HPV test on vaginal self-samples and urine compared to cervical samples (gold-standard) in women referred to colposcopy for an abnormal cervical cytology. At the time of enrolment, a very high rate (75.1%) of women were positive for at least 1 of the 28 HPV genotypes in cervical sample, as expected in a colposcopy setting. This HPV positivity rate is similar to other percentages reported in previous studies conducted on a population with the same characteristics [30,31,32]. However, the inclusion of low-risk HPV types is not recommended within the context of screening as it is not predictive of CIN3 or worse [33]. Consequently, in this study, analysis of concordance had to take only the 14 hr-HPV types into consideration.

Positivity for at least one of the hr-HPV genotypes was found in 65.7% of women, with the most common genotypes being HPV16 and HPV31. Analyzing self-taken specimens provided similar results, with a percentage of 69.4% for HPV-positive women and 65.7% in vaginal and urine samples, and the agreement with cervical swab results was 95.5% (k = 0.898) for vaginal samples and 91.4% (k = 0.764) for urine samples. These promising results are in accordance with previous studies that have evaluated self-collected samples using molecular methods for HPV detection [17,34,35,36]. High-risk HPV detection in urine has been demonstrated to be a good alternative in screening and the results are comparable to those obtained from cervical samples. Van Keer et al. have reported that hrHPV-DNA using the Abbott real-time high-risk HPV assay on home-collected first-void urine has a similar accuracy rate for detecting CIN2+ compared to cervical samples taken by a clinician [35]. The same group also demonstrated that the BD Onclarity HPV assay on first-void urine has a similar clinical sensitivity rate and somewhat lower specificity to detect cervical precancer as compared to clinician-collected cervical samples in a study conducted following the VALHUDES protocol [36,37]. Moreover, since HPV16 and HPV18 are responsible for over 70% of the cases of cervical cancer, the very good concordance between self-collected and cervical specimens is very promising for the introduction of self-collection practice in cervical cancer prevention programs. The slightly inferior agreement between cervical and self-collected samples found in lr-HPV detection (vaginal self-sample: 88.2%, k = 0.762; urine: 85.7%, k = 0.715) may reflect the different distribution of lr-HPV genotypes in the urogenital tract. The different rates of multiple hr-HPV infections detected in cervical and self-collected samples may be caused by the different anatomic sites of specimens’ collection, but it is not well established if the presence of multiple infections could be a risk factor of carcinogenesis or if it is just the detection of HPV DNA of transient infections not associated with lesion development [38]. Our results confirmed that, as compared to clinician-collected cervical samples, urine and self-taken vaginal swabs demonstrated high concordance in agreement with what has been reported in a recent meta-analysis on self-collected samples, which are similarly accurate as clinician samples if tested with HPV assays based on PCR [14].

Moreover, this study demonstrated that the hr-HPV testing of self-collected samples using Anyplex™II HPV28 (Seegene, Seoul, Republic of Korea) is not inferior to the gold standard for the detection of cervical intraepithelial lesions that are potentially at risk of progression. Relative sensitivity rates for the detection of CIN2+ were 1.00 and 0.95 for vaginal self-samples and urine samples, respectively. The relative specificity was 0.89 for self-collected vaginal samples and 0.95 for urine samples based on the total population. From the analysis of women with histological outcomes, the relative specificity was 1.00 for both types of self-collected samples. A good clinical sensitivity rate for the detection of CIN2+ lesions was obtained using the three sample types; even the analysis was restricted to the seven hr-HPV samples described as mainly responsible for cervical cancer development (HPV16, 18, 31, 33, 45, 52, and 58) [39,40], underlining the key role played by such genotypes in the development of cervical cancer.

The high HPV positivity rate in this population and the presence of transient HPV infections that spontaneously regress are responsible for the low specificity rates for the detection of CIN2+ lesions for the three sample types which were demonstrated to be higher when the population examined was constituted by women undergoing screening [41].

Indeed, a limitation of this study is that, in order to achieve statistical power, it was conducted in a population of women referred to colposcopy due to previous cytological abnormalities, as indicated by the VALHUDES protocol [37]. Such a population does not completely reflect that of a screening setting which is mostly composed of healthy women and where the percentage of women with CIN2+ lesions is lower. However, as reported by Arbyn et al., the relative accuracy of hrHPV assays on self-samples versus clinician samples does not vary substantially by clinical setting, and therefore it is possible to estimate overall relative sensitivity and specificity rates under the condition of separating hrHPV assays based on signal amplification from hrHPV assays based on validated polymerase chain reactions [42].

Another important consideration concerns the choice of HPV test to be used on self-samples. In the present study, we selected the Anyplex™ II HPV28 Detection (Seegene, Seoul, Republic of Korea) Kit in order to evaluate the distribution of both lr-HPV and hr-HPV in the different anatomical sites. However, as previously recommended, cervical cancer screening should not include testing for low-risk HPV types [33]. Moreover, to ensure that clinical specificity remains suitably elevated, thereby mitigating the risk of undue false positives, Kim and colleagues suggested cutoff value optimization when reporting HPV infections using the Anyplex assay (≥ 2+; medium viral loads) in a screening setting [43].

A specific study design, the VALHUDES protocol, has been proposed for the combined validation of the HPV test and the self-collection device to establish the proper assay cut-offs and achieve clinical accuracy in providing non-inferior self-collection respect to the cervical specimen [37]. 

Finally, present triage guidelines for hr-HPV-positive women are based on cervical cytology. As cervical cytology cannot be performed on self-collected samples, it is important to consider that in a screening program based on self-sampling, hrHPV-positive women need to be recalled in order to collect an additional cervical sample to perform cytology triage. This introduces an inherent risk of loss associated with the follow-up of HPV-screen-positive women, which may be further exacerbated among non-attendant individuals, a group already predisposed to challenges in adherence. In the future, it would be important to identify new molecular biomarkers suitable for the triage of hr-HPV-positive women, such as methylation- and genotype-specific viral loads, which would allow triage to be performed directly on self-collected samples.

## 5. Conclusions

These results confirmed that performing Anyplex™II HPV28 (Seegene, Seoul, Republic of Korea) on urine and self-collected vaginal samples for the detection of hr-HPV in women with CIN2+ is comparable with that of clinician-taken cervical samples. These findings support initiatives to implement self-sampling as an option to improve participation of non-attending women in screening programs.

## Figures and Tables

**Table 1:** Clinical data of women enrolled.

Cytology	n (%) Total: 245
HSIL	33 (13.5%)
ASC-H	19 (7.7%)
LSIL	107 (43.7%)
ASC-US	57 (23.3%)
AGC	11 (4.5%)
NILM	18 (7.3%)
**Biopsy/Conization**	**n (%) Total: 60**
Adenocarcinoma	1 (1.7%)
Adenosquamous carcinoma	1 (1.7%)
CIN3	33 (55.0%)
CIN2	9 (15.0%)
CIN1	7 (11.6%)
Negative	9 (15%)

**Table 2:** HPV-positive cervical, vaginal, and urine samples according to cytology.

Cytology Results	n° of Samples	Cervical Samples	Vaginal Self-Samples	Urine Self-Samples
		Hr-HPV-Positive	Hr-HPV-Positive	Hr-HPV-Positive
NILM	18	5 (27.8%)	5 (27.8%)	4 (22.2%)
ASC-US/LSIL	164	107 (65.2%)	112 (68.3%)	108 (65.9%)
AGC/ASC-H/HSIL	63	49 (77.8%)	51 (81.5%)	49 (77.8%)

**Table 3:** HPV-positive cervical, vaginal, and urine samples according to histology.

Histology Result	n° of Samples	Cervical Samples	Vaginal Self-Samples	Urine Self-Samples
		Hr-HPV-Positive	Hr-HPV-Positive	Hr-HPV-Positive
<CIN2	16	11 (68.8%)	11 (68.8%)	11 (68.8%)
CIN2+	44	42 (95.5%)	42 (95.5%)	40 (90.9%)

**Table 4:** Accuracy of vaginal self-sample as compared to cervical sample for HPV detection.

HPV Genotype	TP	TN	FP	FN	PPA % (n)	NPA % (n)	OPA % (n)	k Value (95% CI)
hr-HPV	160	74	10	1	99.4% (160)	88.1% (74)	95.5% (234)	0.898 (0.839–0.957)
HPV16	57	183	4	1	98.3% (57)	97.96% (183)	98.0% (240)	0.945 (0.896–0.993)
HPV18	17	226	2	0	100.0% (17)	99.1% (226)	99.2% (243)	0.940 (0.857–1.000)
Other hrHPV	122	108	13	2	98.4% (122)	89.3% (122)	93.9% (230)	0.877 (0.818–0.937)

TP: true positive; TN: true negative; FP: false positive; FN: false negative; PPA: positive percentage agreement; NPA: negative percentage agreement; OPA: overall percentage agreement.

**Table 5:** The accuracy rates of urine as compared to cervical samples for HPV detection.

HPV Genotype	TP	TN	FP	FN	PPA % (n)	NPA % (n)	OPA % (n)	k Value (95% CI)
hr-HPV	149	73	12	11	93.1% (149)	85.9% (73)	90.6% (222)	0.792 (0.712–0.873)
HPV16	51	183	4	7	87.9% (51)	97.9% (183)	95.5% (234)	0.874 (0.801–0.946)
HPV18	14	227	1	3	82.4% (14)	99.6% (227)	98.4% (241)	0.866 (0.737–0.995)
Other hrHPV	117	104	17	7	94.4% (117)	86.0% (104)	90.2% (221)	0.804 (0.730–0.878)

TP: true positive; TN: true negative; FP: false positive; FN: false negative; PPA: positive percentage agreement; NPA: negative percentage agreement; OPA: overall percentage agreement.

**Table 6:** Sensitivity and specificity rates for the detection of CIN2+ in cervical, vaginal, and urine specimens according to hr-HPV test on all women.

CIN2+ Detection				
	Sensitivity % for CIN2+ Detection (95% CI)	Specificity % for <CIN2 Detection (95% CI)	Relative Sensitivity to Cervical Samples for CIN2+ Detection (95% CI)	Relative Specificity to Cervical Samples for <CIN2 Detection (95% CI)
**Cervical Sample**	95.5% (89.3–100.0%)	40.8% (34.0–47.6%)	-	-
**Vaginal Self-Sample**	95.5% (89.3–100.0%)	36.3% (29.7–43.0%)	1.00 (1.00–1.00)	0.89 (0.82–0.97)
**Urine Sample**	90.9% (82.4–99.4%)	39.8% (3.0–46.6%)	0.95 (0.89–1.02)	0.98 (0.87–1.09)

TP: true positive; TN: true negative; FP: false positive; FN: false negative.

**Table 7:** Sensitivity and specificity rates for the detection of CIN2+ in cervical, vaginal, and urine specimens according to the hr-HPV test on 60 women with histological results.

CIN2+ Detection				
	Sensitivity % for CIN2+ Detection (95% CI)	Specificity % for <CIN2 Detection (95% CI)	Relative Sensitivity to Cervical Samples for CIN2+ Detection (95% CI)	Relative Specificity to Cervical Samples for <CIN2 Detection (95% CI)
**Cervical Sample**	95.5% (89.3–100.0%)	31.3% (8.5–584.0%)	-	-
**Vaginal Self-Sample**	95.5% (89.3–100.0%)	31.3% (8.5–55.0%)	1.00 (1.00–1.00)	1.00 (1.00–1.00)
**Urine Sample**	90.9% (82.4–99.4%)	31.3% (8.5–54.0%)	0.95 (0.89–1.02)	1.00 (1.00–1.00)

TP: true positive; TN: true negative; FP: false positive; FN: false negative.

## Data Availability

Final study datasets generated by the study are stored locally and securely at the University of Milano-Bicocca. Anonymized data will be available by request to the corresponding author on a case-by-case basis pending approval by the University of Milano-Bicocca.
